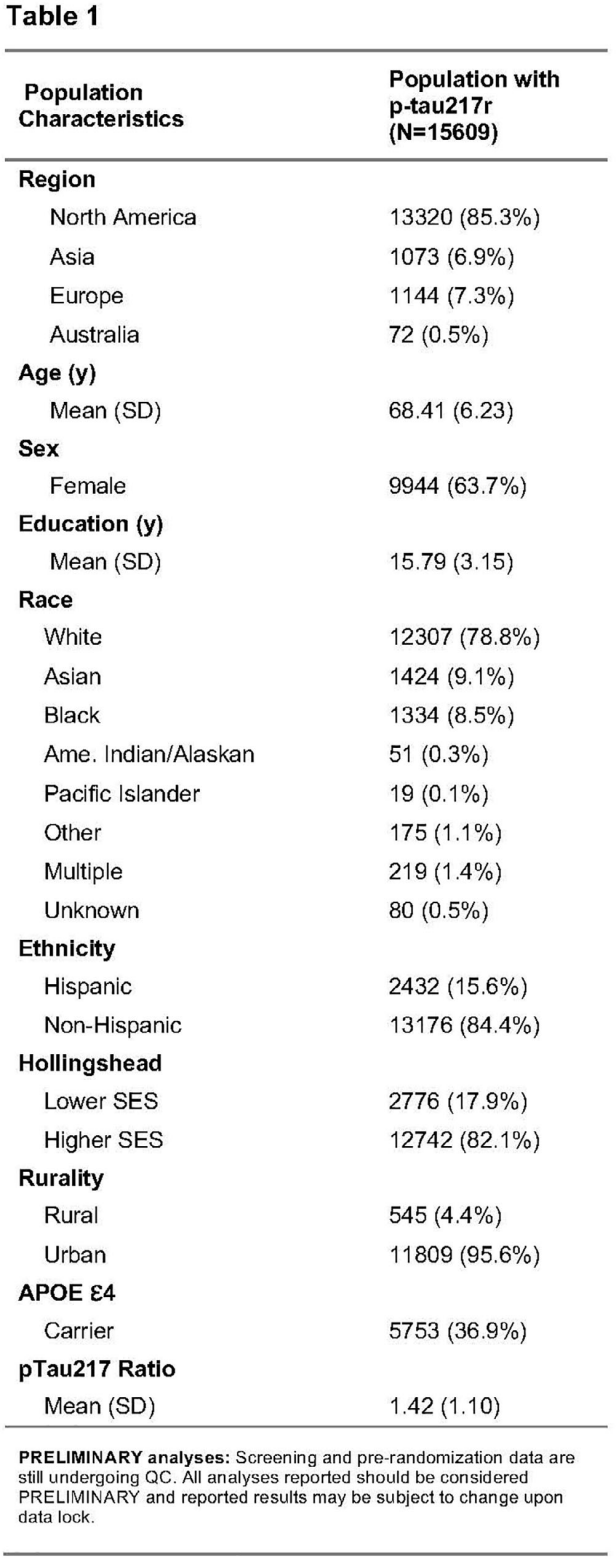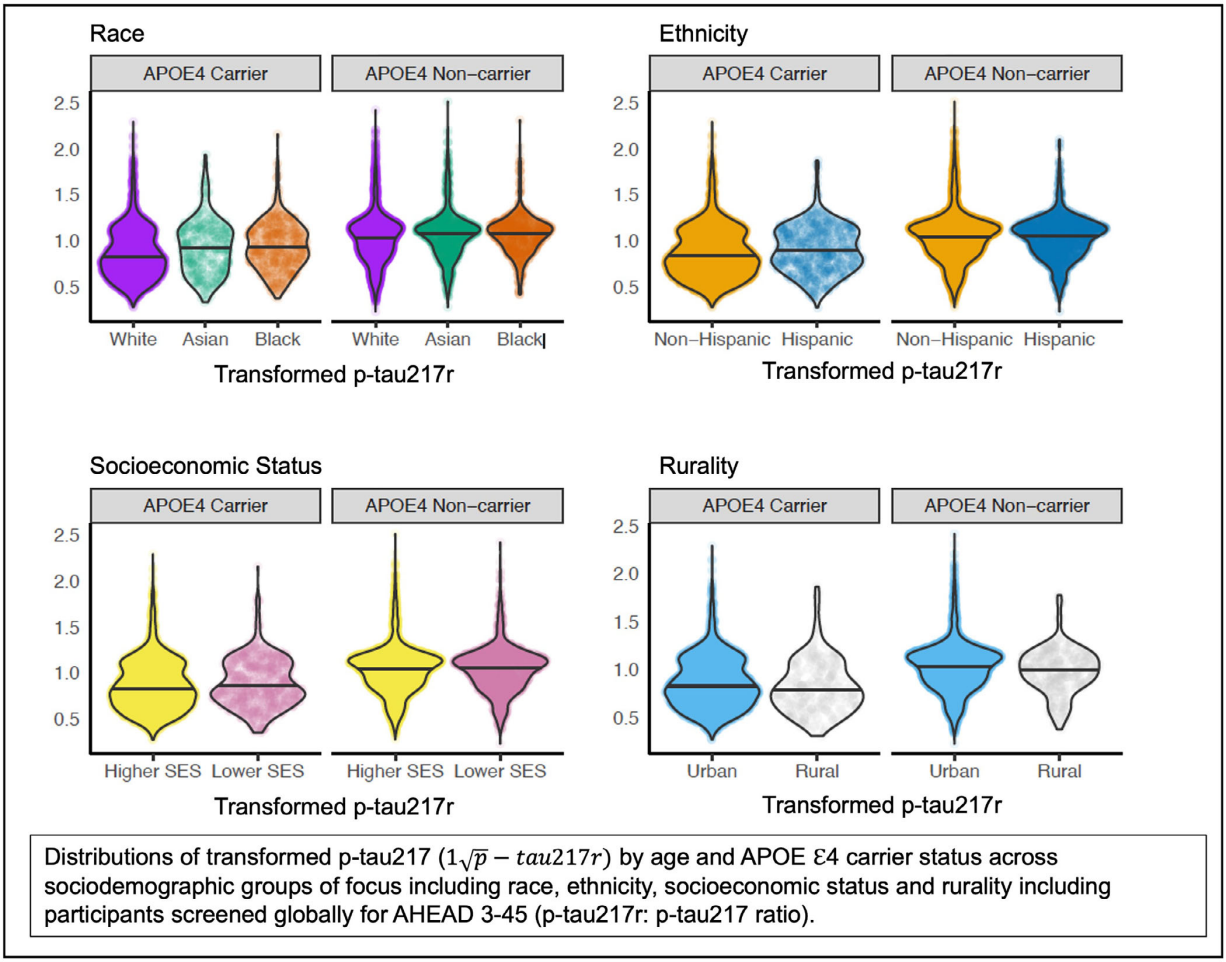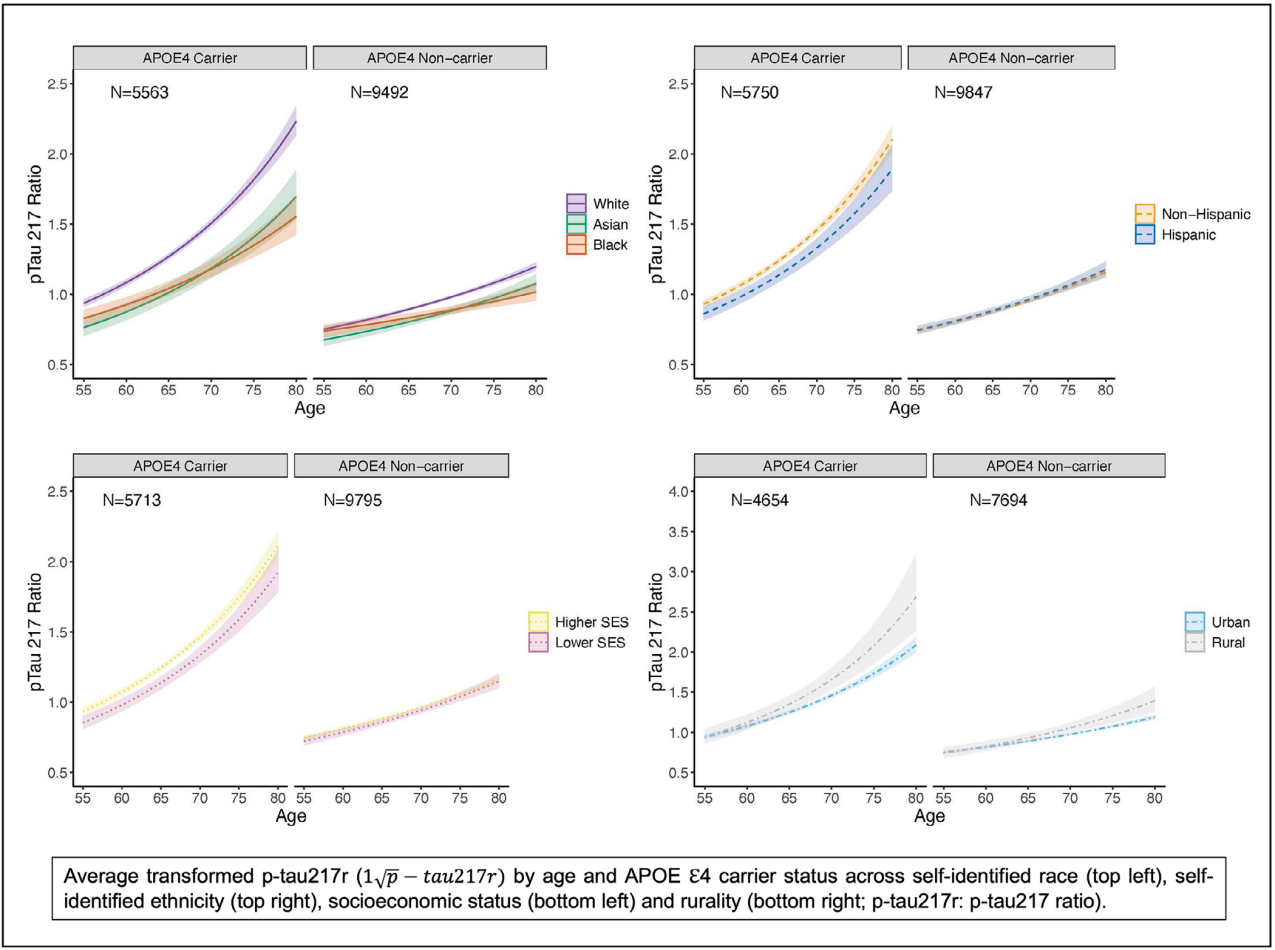# Variations in Plasma *p* ‐tau217 by Sociodemographic Factors Across World Regions in a Preclinical AD Clinical Trials Program: the AHEAD 3‐45 Study

**DOI:** 10.1002/alz70861_108909

**Published:** 2025-12-23

**Authors:** Doris P Molina‐Henry, Rema Raman, Andy Liu, Oliver Langford, Joel B. Braunstein, Shobha Dhadda, Michael C. Irizarry, Joshua D Grill, Keith A. Johnson, Robert A. Rissman, Paul S. Aisen, Reisa A. Sperling, the AHEAD 3‐45 Study Team

**Affiliations:** ^1^ Alzheimer's Therapeutic Research Institute, University of Southern California, San Diego, CA USA; ^2^ C2N Diagnostics, LLC, St. Louis, MO USA; ^3^ Eisai Inc., Nutley, NJ USA; ^4^ Eisai Inc., Woodcliff Lake, NJ USA; ^5^ Institute for Memory Impairments and Neurological Disorders, University of California, Irvine, Irvine, CA USA; ^6^ Massachusetts General Hospital, Brigham and Women’s Hospital, Harvard Medical School, Boston, MA USA; ^7^ Massachusetts General Hospital, Brigham and Women’s Hospital, Harvard Medical School, Boston, MA USA

## Abstract

**Background:**

The AHEAD 3‐45 Study, a preclinical Alzheimer’s Disease (AD) trials program testing lecanemab in asymptomatic individuals with biomarker evidence of disease, completed its randomization October 2024. We have previously reported differences in amyloid abnormalities, as measured by plasma *p* ‐tau217 ratio (*p* ‐tau217r), by race and ethnicity in North America (NA). In these analyses, we report our findings on associations of *p* ‐tau217r by age and APOE ℇ4 carrier status across sociodemographic characteristics in participants from all world regions.

**Method:**

Sociodemographic characteristics included self‐reported race (Asian, Black and White), self‐reported ethnicity (Hispanic and Non‐Hispanic), SES based on Hollingshead score (low‐mid and mid‐high) and rurality defined by Rural‐Urban Commuting Areas (rural/urban). Individuals were screened across 97 clinical sites in 4 world regions (including 7 countries Australia, Canada, Japan, Singapore, Spain, United States, United Kingdom). *p* ‐tau217r was transformed (1√*p*‐tau217r) to meet model assumptions. Plasma *p* ‐tau217r were determined using C2N mass spectrometry for *p* ‐tau217/non‐phosphorylated‐tau. To assess group associations with *p* ‐tau217r, linear models were fitted that adjusted for APOE ℇ4 carrier status, age and all 2‐ and 3‐way interactions.

**Results:**

Demographic characteristics are depicted in Table 1. Figure 1 shows the distribution of *p* ‐tau217r by sociodemographic characteristic. Notably, *p* ‐tau217r increases with age across all groups. APOE ℇ4 carriers demonstrated differences in *p* ‐tau217r across race (*p* <0.001), ethnicity (*p* <0.001) and SES (*p* =0.02) with consistently higher levels of *p* ‐tau217r at younger ages in Whites, non‐Hispanics, and higher SES (Figure 2). There was no effect of rurality on the relationship of *p* ‐tau217r by age and APOE ℇ4 carrier status (*p* =0.58).

**Conclusions:**

In this expanded global cohort, our results are consistent with our previous findings in NA suggesting an effect of race and ethnicity on *p* ‐tau217r among APOE ℇ4 carriers. This finding may reflect underlying differential group‐level prevalence of amyloid pathology through the amyloidogenic modulation of APOE ℇ4. Importantly, our analyses suggest an effect of SES among APOE ℇ4 carriers. Our discussion will examine intersectionality of race, ethnicity and SES. Furthermore, we will report on the relationship of *p* ‐tau217r and amyloid PET to establish whether *p* ‐tau217r displays similar predictive capacity as amyloid PET across groups in this global cohort.